# Genome-wide identification and expression analysis of TCP transcription factors in *Chrysanthemum indicum* reveals their critical role in the response to various abiotic stresses

**DOI:** 10.1186/s12870-025-06521-x

**Published:** 2025-05-13

**Authors:** Shengyan Chen, Bin Chen, Xingnong Xu

**Affiliations:** 1 Yancheng Third People’s Hospital, Yancheng Jiangsu, the affiliated hospital of Jiangsu Vocational College of Medicine, Yancheng, Jiangsu 224008 China; 2 College of Landscape Architecture, Northeast Forestry University, Haerbin, Heilongjiang 150040 China

**Keywords:** *Chrysanthemum indicum*, TCP, Collinearity analysis, Expression profile analysis, Abiotic stress

## Abstract

*Chrysanthemum indicum* is an important medicinal plant that has a particularly strong effect on bacteria and viruses and has antioxidant, anti-inflammatory, and immunomodulatory properties. The genes of the *TCP* family, a group of plant-specific transcription factors (TFs), have been found to play a crucial role in the regulation of plant growth and development as well as resistance to abiotic stress. Nevertheless, no systematic analysis of the *TCP* family genes in *C. indicum* has been performed so far. In the present study, a total of 26 non-redundant *CiTCP* genes were identified in the genome of *C. indicum*. The *TCP* genes were categorized into three subgroups on the basis of the phylogenetic analysis: 7, 9, and 10 genes belonged to the CIN subgroup, CYC/TB1 subgroup, and PCF subgroup, respectively. All *CiTCPs* were unevenly distributed across the 9 chromosomes. *TCP* genes in the same subgroup showed similar gene structures and conserved motifs. Gene duplication analysis revealed that segmental duplications had a significant effect on the expansion of *CiTCP* genes. The analysis of *cis*-elements revealed that *CiTCP* genes may be involved in the regulation of plant development, hormone response and response to abiotic stress. Expression profile analysis of the transcriptome data indicated that *CiTCP* genes exhibited similar or distinct expressions within different tissues and under different abiotic stresses. According to the results of quantitative RT-PCR (qRT-PCR), the expression of 15 selected genes responded strongly to various abiotic stress factors. The results of our studies could provide comprehensive insights into the *TCP* family genes of *C. indicum* for further functional investigations.

**Supplementary Information:**

The online version contains supplementary material available at 10.1186/s12870-025-06521-x.

## Introduction

The Teosinte Branched1/Cycloidea/Proliferating Cell Factors (TCPs) are a group of plant-specific genes that encode TFs (transcription factors) with a TCP domain [1], and were named after the first four identified members: TEOSINTE BRANCHED1 (TB1) from *Zea mays*, CYCLOIDEA (CYC) from *Antirrhinum majus*, PROLIFERATING CELL NUCLEAR ANTIGEN FACTOR 1 and 2 (PCF1 and PCF2) from *Oryza sativa* [2–4]. These members were characterized by the TCP domain, a highly conserved 59-amino acid basic helix-loop-helix (bHLH) structure at the N-terminus that is involved in DNA binding, protein–protein interaction and facilitating nuclear localization [5]. According to their conserved domain, TCP proteins can be divided into two classes: Class I (represented by PCF proteins) and Class II (represented by CYC and TB1) [6]. The most obvious difference between the two subfamilies was that class I members lack 4 amino acids in the basic region of the TCP domain. TCP members belonging to Class II can be further divided into two subclades: CIN and CYC/TB1 [1]. Most of the members of Class II contain a conserved R domain, which may be responsible for facilitating protein–protein interaction [7].

Previous studies showed that Class I genes have generally been associated with cell division and proliferation, whereas Class II genes have mainly been involved in plant stress resistance and lateral organ development [8, 9]. Class I-members have been shown to be involved in the regulation of seed germination [10], flowering [11], stem elongation [12], stamen filament elongation [13] and various hormone signaling pathways in *Arabidopsis thaliana*. *TCP14* and *TCP15* have been found to mediate GA-dependent activation of the cell cycle during germination and to regulate cell proliferation. In tomato, *SlTCP12*, *SlTCP15* and *SlTCP18* were preferentially expressed in the tomato fruit, suggesting a role during fruit development or ripening [14]. In Class II, the famous member *TB1*, has been found to participate in the determination of maize axillary meristem fate [3]. *AtTCP18* and *AtTCP12*, were involved in suppressing bud outgrowth [15]. AtTCP1, a homologous gene of CYC, has been demonstrated to affect plant growth/development by regulating the expression levels of *DWARF4*, a brassinosteroid (BR) biosynthesis gene [16]. *SlTCP9* and *SlTCP7* have similar functions in the initiation and growth of axillary buds [17].

Although much of the research on *TCP* transcription factors has focused on plant growth and development and morphogenesis, in recent years there has been increasing evidence that TCP transcription factors also play an important role in plant responses to environmental stresses. *TCP* transcription factors regulate abiotic stress responses mainly by modulating hormone signaling, ROS scavenging, and stress-responsive gene expression. For instance, under high salt conditions, *Arabidopsis* transferred with *PeTCP10* improved the antioxidant capacity of transgenic *Arabidopsis* plants by promoting catalase (CAT) activity and enhanced their tolerance to H_2_O_2_ compared with wild-type *A. thaliana* [18]. The heterologous expression of the *rice* gene *OsTCP19* in *A. thaliana* has been shown to decrease water loss and the levels of reactive oxygen species in the plants. Additionally, this genetic modification enhances the accumulation of lipid droplets, thereby improving the stress tolerance of transgenic plants during both the seedling and mature stages of development [19]. Overexpression of *ZmTCP42* in *A. thaliana* has been demonstrated to alter seed germination hypersensitivity to ABA and enhances its drought tolerance [20]. The *GmTCP4* transcription factor plays an important role in signaling pathways such as ABA and ET, and the overexpression of *GmTCP4* can increase the drought tolerance in *soybean* [21]. Above all, the *TCP* transcription factor family can actively participate in the plant response to abiotic stresses.

With the rapid development of genome sequencing technology, genome-wide identification and analysis of gene families have become common. To date, the *TCP* gene family has been identified in many different plant species. In eudicots, 24 *TCP* genes have been identified in *Arabidopsis* [22], 30 in *tomato* [14], 19 in *strawberry* [19], 34 in *Pyrus bretschneideri* [23], 36 in *Populus trichocarpa* [24], 31 in *potato* [25]. Among monocots, a total of 28, 66, 42, and 16 *TCPs* have been identified in *Oryza sativa*, *Triticum aestivum*, *Panicum virgatum*, and *Phyllostachys edulis*, respectively [26–29]. However, little is known about the *TCP* gene family in *C. indicum.*

*C. indicum* is an important germplasm resource for perennial herbs of the *Asteraceae* family. The main chemical components of the secondary metabolites are flavonoids, terpenoids and phenolic compounds, which have anti-inflammatory, antioxidant and immunoregulatory properties, so, the medicinal value of *C. indicum* is relatively high. Besides, *Chrysanthemum* is a traditionally famous flower in China, with rich colors and various shapes. It is widely used in landscaping and has high cultural value, ornamental value and economic value. *C. indicum* grows normally in the wild and is often affected by abiotic factors such as drought and low temperature during its growth, which greatly affect yield and quality. However, current research on *C. indicum* focuses on cultivation, breeding, chemical formation, genetic characteristics and medicinal value, and little is known about resistance to abiotic stress. Therefore, the study of the molecular regulatory mechanism of resistance to abiotic stress is of great importance for the breeding of new chrysanthemum cultivars. Unfortunately, systematic investigations of the *TCP* gene family in *C. indicum* have not been reported so far. Recently, the entire genome of *C. indicum* was sequenced [30], laying the foundation for identifying *TCP* genes in *C. indicum*. In the present study, 26 *CiTCPs* were identified. A systematic analysis and prediction on the structures and functions of *TCPs* was performed, including their physicochemical properties, phylogenetic relationships, gene structure, conserved motifs, chromosome locations, gene duplications, promoter *cis*-acting elements, and expression profiles of *CiTCPs* in different tissues and under different abiotic stresses. The results of this study provide a theoretical basis for further research into the potential functions and regulatory mechanisms of *CiTCP* genes in response to abiotic stress.

## Materials and methods

### Identification of *TCP *transcription factors in *Chrysanthemum indicum*

The whole genome data and annotation files were downloaded from the public website (https://figshare.com/projects/Chrysanthemum_indicum_genome_diploid/197683) [30]. To identify all potential *TCP* members in *C.indicum*, the TCP domain HMM profile (accession number PF03634) was downloaded from the InterPro database (https://www.ebi.ac.uk/interpro/) and then used as a query to perform an HMMER search against the *C.indicum* genome database using HMMER 3.0 with the threshold expectation value set to 1e- 20 [31]. All AtTCP and OsTCP proteins were then used as queries to search the *C.indicum* protein database using the BLASTP program with the default parameters [32]. Each matching sequence was then submitted to SMART (http://smart.embl-heidelberg.de/), Pfamscan and NCBI conserved domain search (https://www.ncbi.nlm.nih.gov/Structure/cdd/wrpsb.cgi) to find the TCP domain. The sequences containing no or incomplete TCP domains were manually eliminated. The physicochemical protein properties of the CiTCPs were analyzed using the online website ExPASy (https://web.expasy.org/protparam). Prediction of the subcellular localization was performed using Softberry (http://www.softberry.com/).

### Sequence alignment and phylogenetic analysis of *CiTCP* genes

Multiple protein sequences of *CiTCPs, A.thaliana, O.sativa* and *P. trichocarpa* were aligned using ClustalX 2.0 with default parameters. An unrooted phylogenetic tree based on the full-length protein sequence alignments was constructed with MEGA X software using the maximum likelihood (ML) method with the optimal replacement and 1000 bootstrap replications. The constructed phylogenetic tree was optimized using the online tool Evolview (http://www.evolgenius.info/evolview/#/). The TCP domain of all CiTCPs was visualized using the website Web logo online (http://weblogo.berkeley.edu/logo.cgi).

### Chromosomal distribution and collinearity analysis

The genomic coordinates of the *CiTCPs* were extracted from the genome annotation files. The gene densities of the entire chromosomes were determined and visualized by MapGene2 Chrom (http://mg2c.iask.in/mg2c_v2.1/). The members of the different subgroups on the chromosomes are marked with different colors. Tandem duplicated genes were identified by their physical position on the individual chromosomes. Two or more genes located on the same chromosome were spaced 200 kb apart and showed more than 70% identity when analyzed with BLASTP, which can be defined as tandem duplication events. Segmental duplication events were identified using Multiple Collinearity Scan toolkit (MCScanX) (https://github.com/wyp1125/MCScanX) with default parameters [33]. The Circos program was used to draw collinearity maps to represent duplicated gene pairs [34]. The Ka/Ks calculator 2.0 was used to calculate the ratio between the non-synonymous rate (Ka) and the synonymous substitution rate (Ks) of duplicated genes [35].

### Analysis of gene structures, conserved motifs and *cis*-element analysis of *CiTCP* genes

The corresponding CDS and DNA sequences of the candidate *TCP* genes in *C. indicum* were also retrieved from the genome database. The intron distributions, positions and phases of the *CiTCP* genes were extracted. Candidate CiTCP protein sequences were analyzed using MEME version 5.5.5 software (https://meme-suite.org/meme/) with the following parameters: maximum motif number was 10; minimum motif width was 6; maximum motif width was 50; and the distribution of motif occurrences was zero or one per sequence. Evolview was used to integrate and visualize images of the phylogenetic tree, gene structures and conserved motifs. The regions upstream of 2000 bp were used to search for regulatory elements.

### Gene Ontology (GO) annotation and Protein–Protein Interaction (PPI) analysis

Gene Ontology (GO) analysis was carried out for the *CiTCP* genes from the EggNOG database (evolutionary genealogy of genes: Non-supervised Orthologous Groups (http://eggnogdb.embl.de/#/app/home). All genes of *C. indicum* served as a reference set. A Gene Ontology (GO) enrichment analysis was performed. TBtools was used to obtain the GO enrichment terms with corrected *p*-values (≤ 0.05) [36]. Statistical analyzes and mapping were performed via the Biozeron Cloud Platform (http://www.cloud.biomicroclass.com/CloudPlatform). The TCP protein sequences were uploaded to the STRING database (https://string-db.org/) for node comparison, and the relationships between major proteins were predicted based on *Arabidopsis* protein interactions.

### Analysis of gene expression patterns of *CiTCPs* using RNA-Seq databases

RNA-Seq data were used to analyze the expression profiles of *CiTCP* genes in different tissues (roots, buds, tongue flowers, leaves, tubular flowers and stems) and under different abiotic stresses (cold, heat, UVB and Cd^2+^). Expression data of fragments-per-kilobase-per-million (FPKM) were retrieved from genome-wide RNA-Seq databases (unpublished). Heat maps were drawn based to gene expression values (FPKM) using the TBtools software.

### Plant material and treatments

The wild type of *C. indicum* used in this study was obtained from the Chrysanthemum Research Center in Cold Land, Northeast Forestry University, Har bin, China (126°63′E, 45°72′N). The seeds that were full and uniform in shape were selected. The samples were then exposed to two layers of filter paper moistened with distilled water in round petri dishes at 25 °C for 3 days. The seedlings were transplanted into square plastic pots with vermiculite in a greenhouse for growth (16/8 h light/dark photoperiod, 60%–70% relative humidity). Four-week-old *C. indicum* seedlings were subjected to different treatments. For cold stress, *C. indicum* seedlings were transferred to an artificial climate chamber at 4 °C. For drought stress, the plants were irrigated with 10% polyethylene glycol (PEG) 6000. For salt stress, 100 mM NaCl was used to simulate salt stress. Leaves were sampled at 0, 0.5, 2, 6, 12 and 24 h, and the samples treated for 0 h were used as controls. Leaf samples from each treatment were collected three times. All samples were immediately stored at − 80 °C prior to RNA extraction.

### RNA isolation and quantitative real-time PCR (qRT-PCR) for *CiTCP* genes

Total RNA was isolated from leaves using the E,Z,N,A,TotalRNAKitI-R6827, according to the manufacturer’s protocol. The *Evo M-MLV* RT Mix Kit with gDNA Clean for qPCR Ver.2 was used to perform the reverse transcription experiment. The quantitative real-time PCR reaction was performed to analyze the relative transcript levels of selected genes with the Light-Cycle 96 instrument using the SYBR Green Premix *Pro Taq* HS qPCR Kit III (Low Rox Plus). The reaction was performed as follows: 95 °C for 30 s, followed by 40 cycles of 95 °C, for 5 s, and 55 °C for 30 s, 72 °C for 30 s. Each reaction was performed in three biological replicates, and relative expression was calculated using the 2 − ^∆∆Ct^ method. The results were analyzed as mean ± SE. The primers used in this study were designed with Primer5.0 and are listed in Additional file 8.

## Results

### Identification of TCP genes in *C. indicum*

A total of 26 *CiTCP* genes were identified in the genome of *C. indicum* by HMMER and local BLASTP searches. These genes were designated as *CiTCP1* to *CiTCP26* based on their chromosomal distribution. Details of the physical and chemical properties of the CiTCP genes are given in Table 1. The length of the CiTCP proteins varied between 154 (CiTCP 11) and 440 (CiTCP 7) amino acid residues. The theoretical isoelectric point (PI) values ranged from 5.55 to 9.99. CiTCP11 showed the lowest molecular weight value (17.01 kDa), while the highest molecular weight (47.50 kDa) was observed for CiTCP7. The predicted subcellular localization revealed that most CiTCP proteins are localized in nuclears (14), while the rest are localized in the extracellular spaces (12).

**Table 1:** The detail information of 26 *CITCP* genes identified in present study

Gene name	Genome ID	Chr	Start	End	Group	ProteinLength(aa)	IsoelectricPoint(pI)	MolecularWeight(Da)	SubcellularLocalization
CITCP1	CI01 AG002770.mRNA1	chr01	56430541	56432036	PCF	258	9.16	27157.25	Nuclear
CITCP2	CI01 AG003584.mRNA1	chr01	84706373	84707355	PCF	241	6.34	26626.14	Nuclear
CITCP3	CI01 AG005204.mRNA1	chr01	1.38E+ 08	1.38E+ 08	CYC/TB1	242	9.99	27659.85	Nuclear
CITCP4	CI02 AG008829.mRNA1	chr02	2.43E+ 08	2.43E+ 08	PCF	375	7.02	40780.89	Nuclear
CITCP5	CI02 AG009025.mRNA1	chr02	2.56E+ 08	2.56E+ 08	CYC/TB1	395	6.24	44600.97	Extracellular
CITCP6	CI03 AG002190.mRNA1	chr03	62148621	62150616	CYC/TB1	386	6.29	43763.01	Extracellular
CITCP7	CI03 AG002748.mRNA1	chr03	82475882	82483796	CIN	440	9.28	47502.98	Extracellular
CITCP8	CI03 AG005180.mRNA1	chr03	2.16E+ 08	2.16E+ 08	PCF	207	6.04	21927.23	Nuclear
CITCP9	CI04 AG006886.mRNA1	chr04	2.13E+ 08	2.13E+ 08	PCF	287	6.26	31017.81	Nuclear
CITCP10	CI06 AG006596.mRNA1	chr06	2.64E+ 08	2.64E+ 08	CIN	401	6.11	43741.33	Nuclear
CITCP11	CI07 AG001210.mRNA1	chr07	25343047	25343713	PCF	154	7.15	17014.4	Nuclear
CITCP12	CI07 AG004238.mRNA1	chr07	1.52E+ 08	1.52E+ 08	CYC/TB1	407	5.55	45712.02	Extracellular
CITCP13	CI07 AG004239.mRNA1	chr07	1.52E+ 08	1.52E+ 08	CYC/TB1	384	8.68	43585.72	Extracellular
CITCP14	CI07 AG006830.mRNA1	chr07	2.85E+ 08	2.85E+ 08	CIN	269	6.86	30497.28	Extracellular
CITCP15	CI07 AG006987.mRNA1	chr07	2.9E+ 08	2.9E+ 08	PCF	385	8.66	41996.34	Nuclear
CITCP16	CI08 AG002435.mRNA1	chr08	51058930	51060744	CIN	339	6.36	37835.16	Extracellular
CITCP17	CI08 AG002612.mRNA1	chr08	57087768	57089369	PCF	371	6.5	39803.76	Nuclear
CITCP18	CI08 AG002934.mRNA1	chr08	66038522	66040513	CYC/TB1	334	9.25	38251.77	Extracellular
CITCP19	CI08 AG003093.mRNA1	chr08	73877393	73880962	PCF	314	9.08	34219.08	Nuclear
CITCP20	CI08 AG003744.mRNA1	chr08	99796280	99798529	CIN	290	9.12	32540.63	Extracellular
CITCP21	CI08 AG003857.mRNA1	chr08	1.06E+ 08	1.06E+ 08	CYC/TB1	291	8.45	32850.9	Extracellular
CITCP22	CI08 AG003858.mRNA1	chr08	1.06E+ 08	1.06E+ 08	CYC/TB1	322	9.02	37273.75	Extracellular
CITCP23	CI08 AG003859.mRNA1	chr08	1.07E+ 08	1.07E+ 08	CYC/TB1	304	6.1	34803.6	Extracellular
CITCP24	CI09 AG001712.mRNA1	chr09	31064460	31075392	CIN	341	6.43	37704.18	Nuclear
CITCP25	CI09 AG002811.mRNA1	chr09	57386093	57387272	PCF	262	9.99	28288.43	Nuclear
CITCP26	CI09 AG008294.mRNA1	chr09	3.28E+ 08	3.28E+ 08	CIN	380	5.95	41649.37	Nuclear

### Phylogenetic analysis and classification of *TCP *members in *C. indicum*

**Fig. 1:**
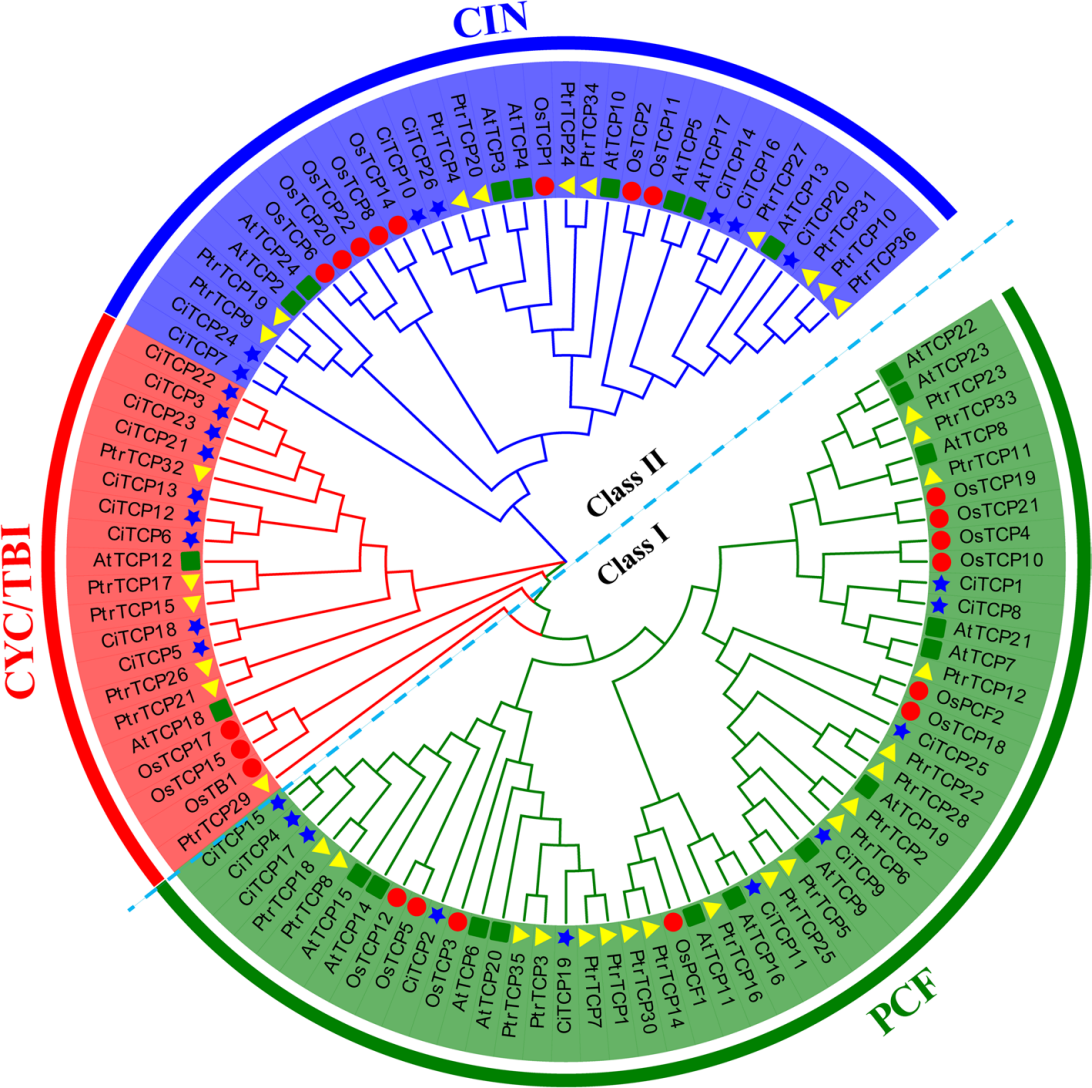
Phylogenetic analysis of TCP transcription factors from *Chrysanthemum indicum*, *Arabidopsis thaliana*, *Oryza sativa* and *Populus trichocarpa*. ClustalW was applied for the alignment of protein sequences. Maximum-likelihood method with 1000 bootstrap replicates was utilized to construct the phylogenetic tree in MEGA X software. Different colors represent different sub-classes in the *TCP* gene family. The *TCPs* from *Chrysanthemum indicum*, *Arabidopsis thaliana*, *Oryza sativa* and *Populus trichocarpa* are marked with blue star, green square, red circle and yellow triangle, respectively

To investigate the evolutionary and phylogenetic relationships among the *C. indicum* TCPs, the full-length amino acid sequences of the TCP members from *C. indicum* (26) and the model species *A. thaliana* (22), *O. sativa* (18) and *P. trichocarpa* (35) were used to construct a phylogenetic tree (Fig. 1). In accordance with the classification of TCPs from other known species, 26 *CiTCPs* can be divided into two subfamilies: Class I and Class II, Class I (PCF) contains 10 members, and 16 members are assigned to Class II which in turn can be divided into two subgroups: CIN (7 members) and CYC/TB1 (9 members). To confirm the reliability of our phylogenetic tree generated by the maximum likelihood (ML) method, we use the neighbor-joining (N-J) method. The result showed that the classification of *CiTCP* genes using the two methods was consistent.

**Fig. 2:**
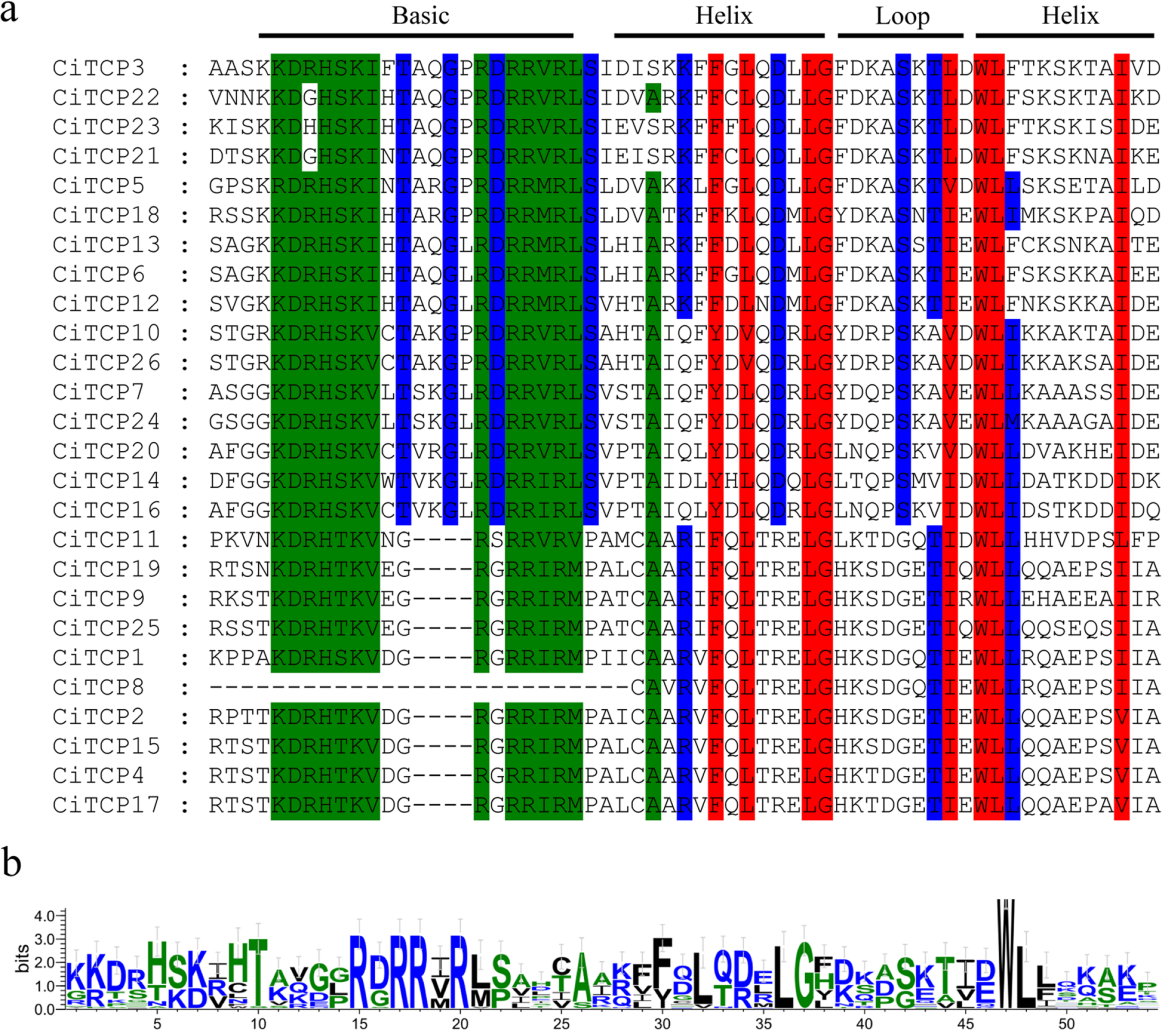
Multiple sequence alignment of CITCPs. **a** Alignment of the TCP domain in all identified TCP TFs; **b** The sequence logo was constructed by Weblogo, the height of each letter representing the corresponding amino acid the frequency of occurrence

To further explore the evolutionary relationships of the *CiTCP* genes, we also performed conserved domain sequence alignment analysis with all TCP protein sequences in *C. indicum.* The results (Additional file 1 and Fig. 2) revealed that all the *CiTCP* genes contained a conserved basic region at the N-terminus and a helix-loop-helix motif at the C-terminus. Consistent with the TCP domain structure in other plant species, the *TCP* members in Class I have four fewer amino acid residues than Class II members in the basic region. These results indicate that the *TCP* gene family, together with other plant species, has a high level of conservation. In addition to the TCP domain, only a few members in Class II have an R domain with 18 amino acid residues. As shown in Additional file 2, eight Class II TCP proteins contained the R domain at the C-terminus of the TCP domain, seven of which were CiTCPs (CiTCP3, CiTCP5, CiTCP6, CiTCP13, CiTCP21, CiTCP22 and CiTCP23) from the CYC/TB1 subgroup, CiTCP24 from the CIN subgroup. At the same time, we found that the R domain was more conserved in the CYC/TB1 subgroup compared to the CIN subgroup.

### Analysis of chromosomal localization and duplication events of *TCP* gene in *C. indicum*

**Fig. 3:**
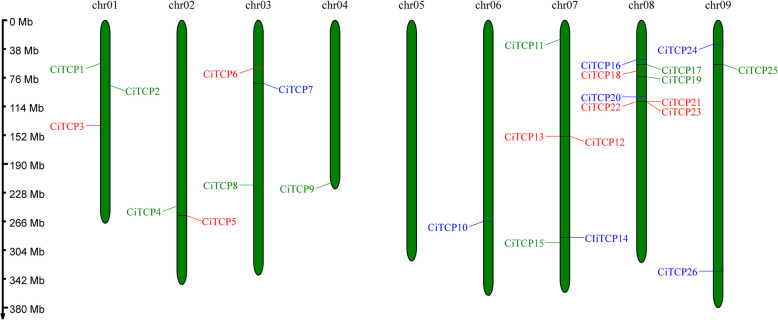
Chromosomal locations of *Chrysanthemum indicum TCP* genes. The chromosomal position of each *CITCP* was mapped according to the genome of *C. indicum*. The chromosome number is marked at the top and the ratio on the left represents the chromosome length with the unit for the scale is mega bases (Mb)

Our initial step was to map all identified *CiTCPs* on the chromosomes (Fig. 3) to investigate the molecular mechanisms behind *C. indicum's* expansion. During the analysis, it was found that 8 out of 9 chromosomes had 26 identified *TCP* genes distributed unevenly, apart from chr05. With the highest proportion of *TCP* genes, Chr08 was the most prominent *TCP* and contained 8 members (30.7%) of the total chromosomes. Chr07 harbored the second largest number of *TCP* genes, with a total of five. There were a small number of *TCP* genes present on chr01, chr03, and chr09, with each having three members. Two members were present in chr02, while chr04 and chr06 each had a single member.

**Fig. 4:**
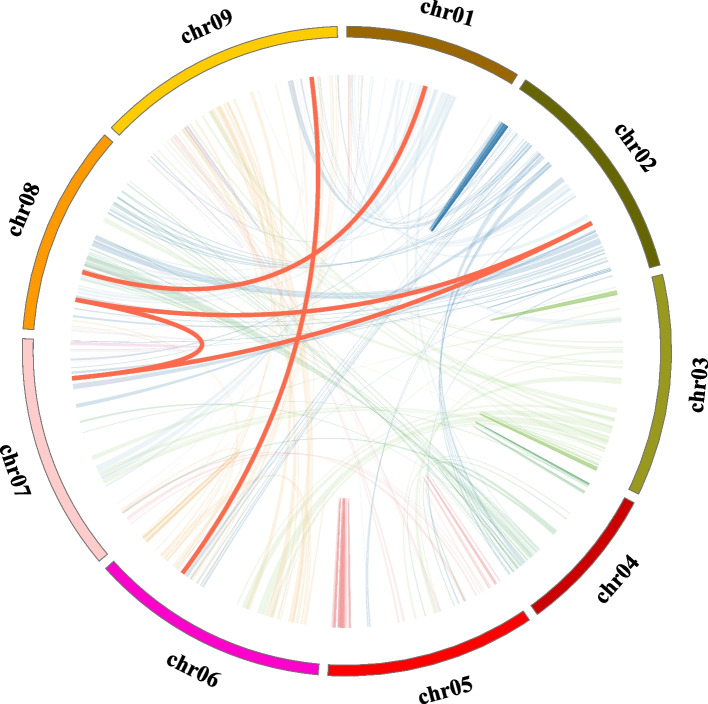
Schematic representation of the inter-chromosomal relationships of *TCP* genes in *C. indicum*. Gray lines in the background indicate the synteny blocks within the whole *C. indicum*, and the redlines denote the segmental duplication *TCP* gene pairs

**Fig. 5:**
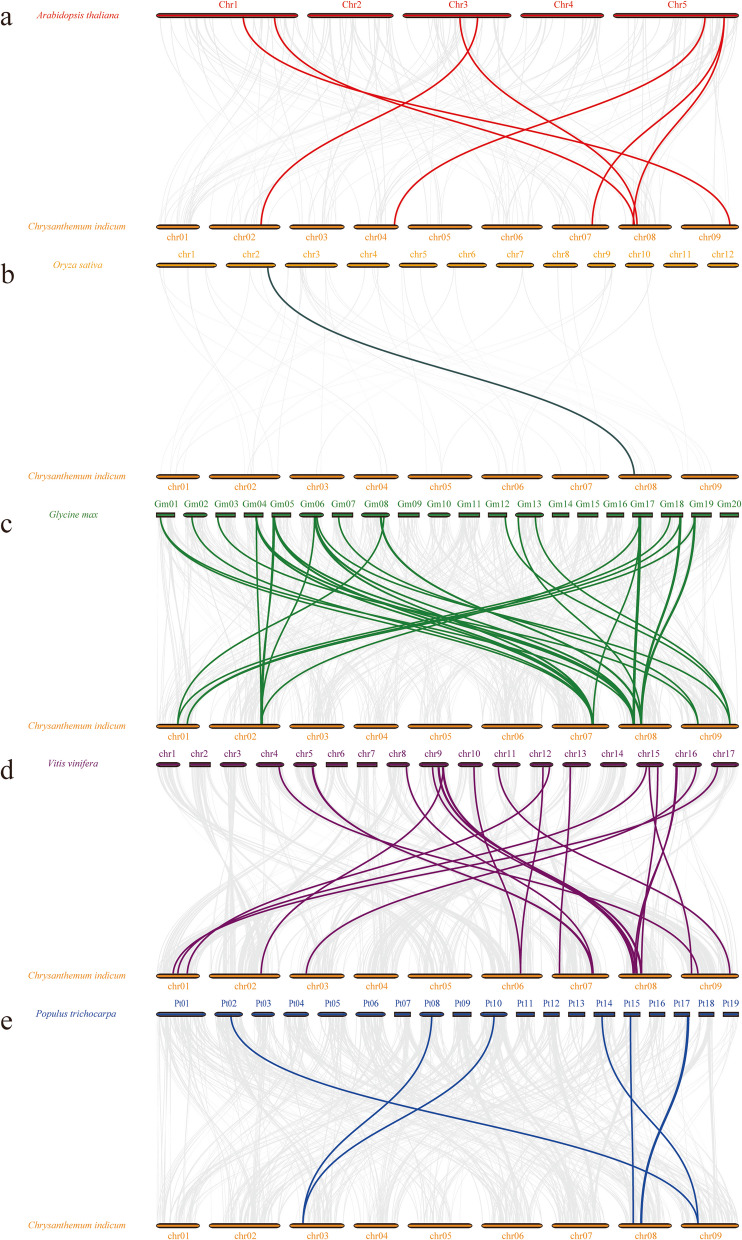
Synteny relationships of *TCPs* in *Chrysanthemum indicum* and five representative species. Synteny relationships of *TCPs* between *Chrysanthemum indicum* with (**a**) *Arabidopsis thaliana*, (**b**) *Oryza sativa*, (**c**) *Glycine max*, (**d**) *Vitis vinifera*, (**e**) *Populus trichocarpa*

A syntenic analysis of the *CiTCP* family genes was performed to gain a more in-depth understanding of their expansion and evolution. The overarching collinearity analysis revealed that five pairs of segmental duplications were identified (Fig. 4), whereas no tandem duplication events were found (Fig. 3), indicating that segmental duplication was the primary driving factor for the expansion of the *TCP* gene family in *C. indicum.* Collinearity maps of the evolutionary relationships among 26 *CiTCPs* between *C. indicum* and *Arabidopsis, G. max*, *P. trichocarpa* and *Vitis vinifera*. According to the syntenic maps (Fig. 5), the *TCP* genes in *C. indicum* had the most homologous gene pairs in *G. max* (39 orthologous gene pairs), followed by *V. vinifera* (24 orthologous gene pairs), *A. thaliana* and *P. trichocarpa* (7 orthologous gene pairs), and fewer homologous gene pairs in *O. sativa* presented 1 orthologous gene pair (Additional file 3). The evolutionary selection pressure on the *CiTCP* family was investigated by calculating the Ka/Ks ratios. The results showed that the Ka/Ks ratio of the duplicated gene pairs was less than 1.0, indicating that the gene pairs were subjected to strong purifying selection pressure after duplication events (Table 2).

**Table 2:** The duplicated genes in *CiTCP*s

Duplicated gene 1	Duplicated gene 2	Ka	Ks	Ka/ks	Purifying Selection	Duplicate Type
CITCP3	CITCP21	0.437364754	1.486320535	0.294260049	Yes	Segmental
CITCP10	CITCP26	0.437364754	1.486320535	0.294260049	Yes	Segmental
CITCP15	CITCP17	0.206590838	0	0	No	Segmental
CITCP4	CITCP15	0.190807194	2.125241646	0.089781411	Yes	Segmental
CITCP4	CITCP17	0.190747637	2.570353397	0.074210666	Yes	Segmental

### Analysis of the gene structure and conserved motifs of the *CiTCP* gene family

**Fig. 6:**
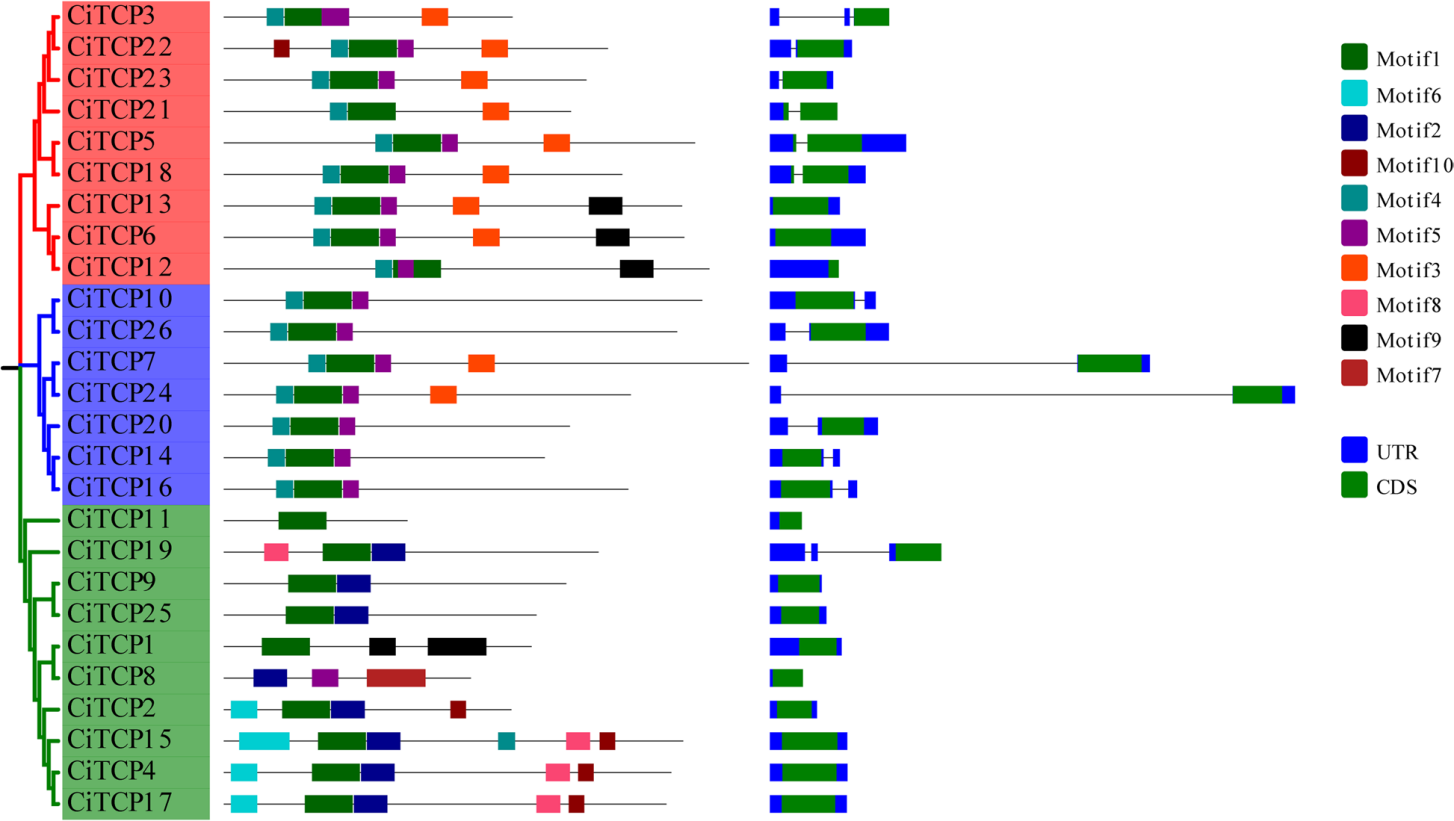
Phylogenetic relationships, gene structure analysis, conserved motifs of *CITCPs*. Details of clusters in the phylogenetic tree are shown in different colors. The left panel represents the motif composition of the *CITCPs*. The right panel shows the intron–exon structure of the *CITCPs*. Different motifs are indicated by different colors. Introns are represented by black lines, exons by green boxes and UTR by blue boxes

To enhance understanding of the evolutionary connections between the various members of the *CiTCP* gene family, a map was created that depicts the phylogenetic tree, exon–intron structure, and conserved motif. The organization of 26 *CiTCP* members is highly conserved and simple, as demonstrated in Fig. 6. The remaining members had only one exon, while three out of 26 *CiTCP* genes had two coding regions (CDS). *CiTCP3* and *CiTCP19* were particularly notable for having two introns, whereas the remaining genes had simple structures that could consist of either one or no introns. All members had one or more UTRs in untranslated regions. The distribution patterns of exons and introns in *CiTCP* genes within the same subgroup mirrored those of their counterparts in terms of length and number, which substantiated the classification of subgroups and evolutionary relationships.

Analysis of possible conserved motif compositions in the *TCP* gene family was performed to gain insight into the diversity and functional properties of the *TCP* genes in *C. indicum*. The MEME program was utilized to identify and annotate the sequences of these motifs, and Additional file 4 contains detailed information about them. As shown in Fig. 6, the highly conserved TCP domain (motif 1) was detected in all CiTCP proteins. The conserved R domain (motif 3) was present in 10 members of Class II (Additional file 4). Also, the N-terminal TCP domain of motif 4 was detected in all class II members. It was found that certain subgroups contain multiple distinctive motifs, including motif 2, motif 6, motif 7, and motif 8, in PCF subgroup proteins according to the results. Although the motif organization of the *TCPs* differed among the different subgroups, it was consistent with the phylogenetic tree, indicating that functional similarity and motif compositions may have contributed to functional divergence in the evolutionary process.

### Analysis of* Cis*-acting elements in the promoters of *CiTCPs*

**Fig. 7:**
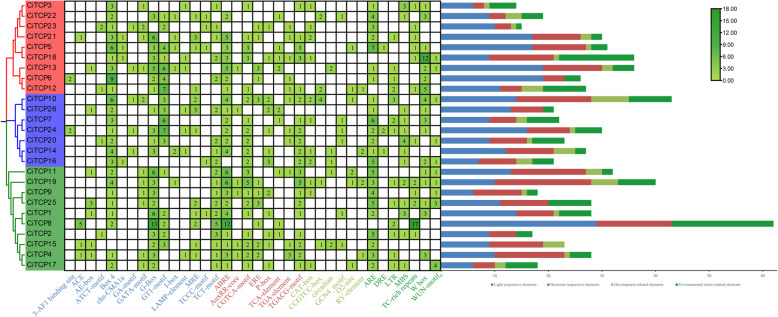
Analysis of *cis*-elements in the promoter of the *CITCP* genes. The phylogenetic tree of 26 *CITCPs* was present on the left. The middle panel showed the heatmap of *cis*-elements in the promoter of the *CITCP* genes. The number of *cis*-elements for each *CITCP* gene in four categories

Differences in the transcript expression and biological function of genes can be revealed by revealing the crucial regions in gene promoters that initiate transcription at transcription factor-binding sites. 26 *CiTCPs* were subjected to *cis*-element analysis in order to obtain more valuable information. The results (Additional file 5 and Fig. 7) showed that 36 *cis*-acting elements, which included light responsive, hormone responsive, development related, and environmental stress-related elements, were identified and categorized into four categories. Light responsive elements, which are frequently found in *TCP* genes, accounted for the highest proportion. The Box 4, G-box, and GT1-motif elements stand out in terms of prominence. The second most distributed element was found to be the hormonal response elements, following the light responsive elements. Except for *CiTCP3*, *CiTCP16*, and *CiTCP23*, all *CiTCPs* had one or more ABREs. The promoters of 20 *CiTCPs* contain the CGTCA and TGACG patterns that are associated with the MeJA response. Numerous elements that respond to auxin (AuxRR-core and TGA-elements), ethylene (EREs), salicylic acid (TCA-elements), and gibberellin (P-boxes) are present in certain *CiTCPs*. Moreover, six types of elements related to plant growth and development were present in 21 members in small numbers, such as the CAT-box in 11 members, and the CCGTCC-box, circadian, GCN4_motif, O2-site and RY-elements in 5, 5, 6, 6 and 4 *CiTCP* genes. Additionally, we discovered seven types of cis-acting elements that cause environmental stress, and ARE elements were the most plentiful (68). The second highest number of W-box elements (46) was found in 17 *CiTCP* genes after that. The presence of MBS elements in half of the members suggests that drought stress may regulate these genes. *CiTCP5*, *CiTCP14*, and *CiTCP24* contain DRE, which is a component of dehydration, low-temperature, and salt stresses. 13 *CiTCP* members had LTRs that were responsible for the low-temperature responsiveness with a number of 18. In 12 genes, there were TC-rich repeats and WUN-motifs that were observed.

### GO enrichment analysis and PPI prediction

**Fig. 8:**
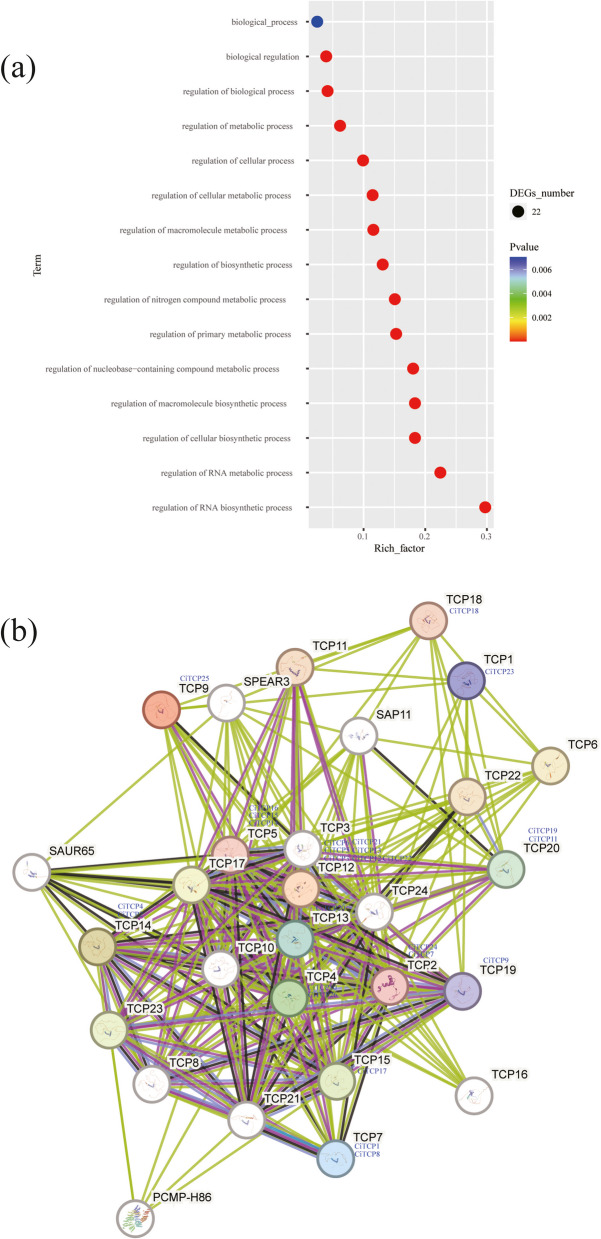
Functional analysis of CiTCPs. **a** GO enrichment analysis of *CiTCP* genes; vertical axis indicates GO terms; horizontal axis indicates Rich factor. the larger the Rich factor, the stronger the enrichment. The size of the dots indicates the number of genes in the GO terms; **b** Protein–protein interaction analysis among CiTCPs by the STRING database. The results were based on an *Arabidopsis* association model

Using gene ontology (GO) to categorize *CiTCPs* into functional groups, it was determined that they were only assigned to one category: biological processes (15 terms) (Fig. 8 and Additional file 6). In the biological process, the 22 genes were found to be associated with RNA biosynthetic process, RNA metabolic process, cellular biosynthetic process, macromolecule biosynthetic process, nucleobase-containing compound metabolic process, primary metabolic process, nitrogen compound metabolic process, biosynthetic process, macromolecule metabolic process, cellular metabolic process, metabolic process, biological process. It can be inferred from these annotations that the *CiTCP* genes are involved in biological processes.

A protein–protein interaction (PPI) network was constructed based on STRING download data to predict the function of the *CiTCP* family genes. As shown in Fig. 8, members in the same subgroup have the same homologous genes, such as, CiTCP3/5/6/12/13/21/22 belong to the CYC/TB1 subgroup have high homology with AtTCP12, CiTCP7/24 belong to the CIN subgroup have high homology with AtTCP14. It should be noted that SAP11, zinc finger AN1 and C2H2 domain-containing stress-associated protein 11, may be responsible for the environmental stress response. According to the map, SAP11 can interact with TCP12/18/4/15/17/5/6/24/20/3/13/14/2/SPEAR3, while CiTCP3/5/6/12/13/21/22 shared homology with AtTCP12, CiTCP18 shared homology with AtTCP18, CiTCP10/26 shared homology with AtTCP4, CiTCP17 shared homology with AtTCP15, CiTCP14/15/16 shared homology with AtTCP5, CiTCP19/11 shared homology with AtTCP20, CiTCP20 shared homology with AtTCP13, CiTCP2/4 shared homology with AtTCP14, CiTCP7/24 shared homology with AtTCP2, interacts with SAP 11. According to the map, SAP11 can interact with TCP12/18/4/15/17/5/6/24/20/3/13/14/2/SPEAR3, while CiTCP3/5/6/12/13/21/22 shared homology with AtTCP12, CiTCP18 shared homology with AtTCP18, CiTCP10/26 shared homology with AtTCP4, CiTCP17 shared homology with AtTCP15, CiTCP14/15/16 shared homology with AtTCP5, CiTCP19/11 shared homology with AtTCP20, CiTCP20 shared homology with AtTCP13, CiTCP2/4 shared homology with AtTCP14, CiTCP7/24 shared homology with AtTCP2, interacts with SAP 11. The above results indicate that CiTCP proteins tend to form protein complexes to exercise their functions, and it is hypothesized that the possible diversity of *CiTCP* gene functions is related to the diversity of interacting proteins. Although the interaction network of TCP proteins in *C. indicum* needs to be further verified, our results provide an important theoretical basis for exploring the molecular mechanism of *TCP* genes in *C. indicum*.

### Tissue specific expression patterns of *CiTCPs*

**Fig. 9:**
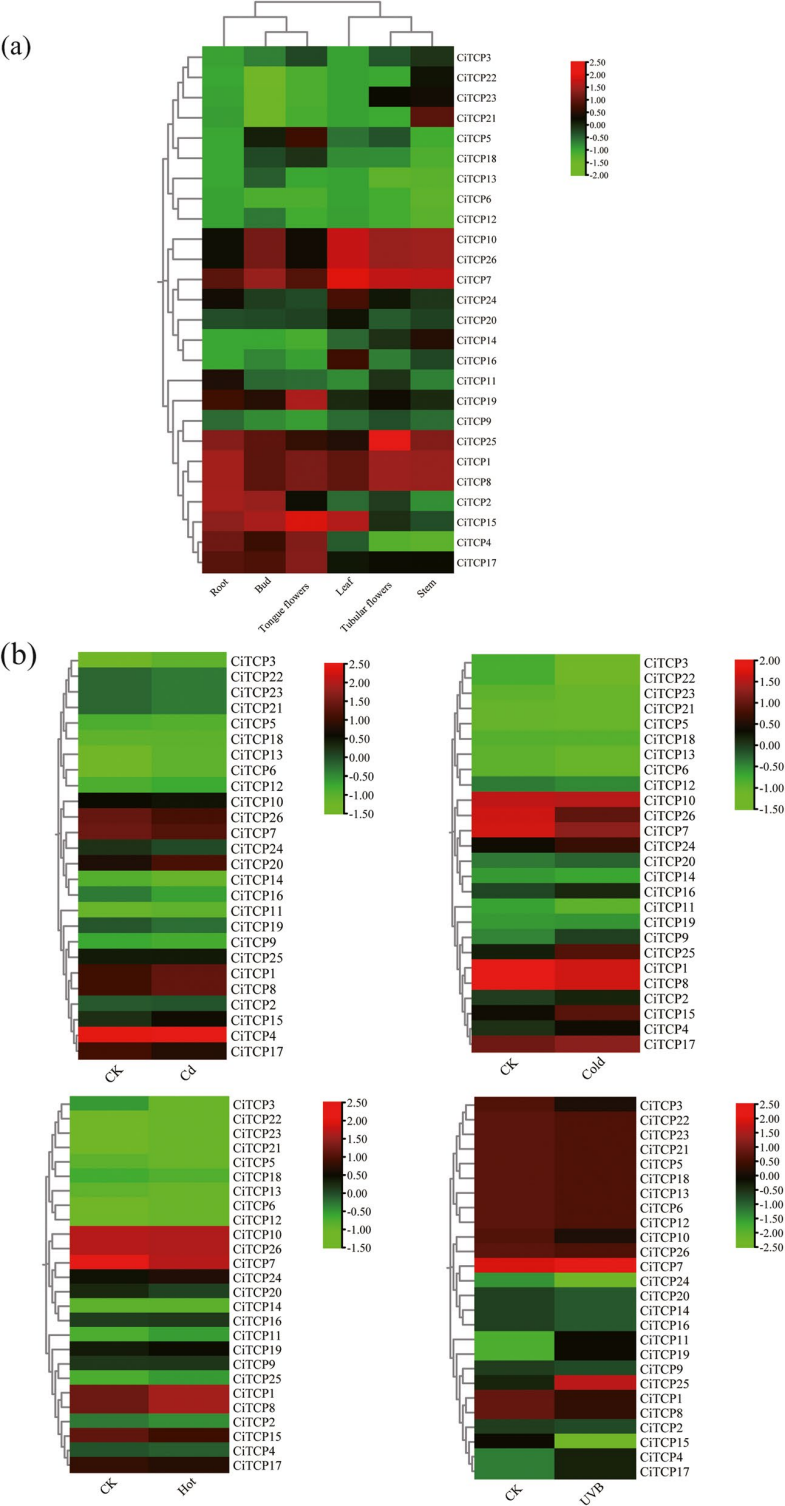
Expression profiles of *CITCPs* in different tissues under normal conditions (**A**), and in response to cold (4 ℃), heat (42 ℃), Cd (200 μmol) and UVB (**B**) from RNA-seq data. Color scale at the right of the heatmap shows the expression level, red indicates high transcript abundance while green indicates low abundance

Tissue-specific expression patterns of all *CiTCP* genes were analyzed using RNA-Seq data to gain a better understanding of their potential roles in *C. indicum* development. The *CiTCP* genes were found to have obvious tissue specificity in the results, as shown in Additional file 7 and Fig. 9. The expression of all *CiTCP* genes was detected in least one tissue, in which 12, 11, 10, 11, 13 and 13 genes presented high transcript abundance (FPKM > 5) in the bud, leaf, tubular flowers, tongue flowers, root, stem tissues, respectively. *CiTCP* genes, such as *CiTCP1*, *CiTCP7*, *CiTCP8*, *CiTCP10*, *CiTCP17*, *CiTCP25*, and *CiTCP26*, have identical expression profiles in different organs and tissues, as demonstrated by Fig. 9. These genes, which are ubiquitously high expressed in all tissues, include *CiTCP1*, *CiTCP7*, *CiTCP8*, *CiTCP10*, *CiTCP17*, *CiTCP25*, and *CiTCP26*. Alternatively, other *CiTCPs* exhibited tissue-specific transcript accumulation patterns, which could suggest the functional diversification of *CiTCP* genes during growth and development. For example, *CiTCP3*, *CiTCP6*, *CiTCP9*, *CiTCP12*, *CiTCP13*, *CiTCP16*, *CiTCP18* were expressed at a very low level in all tested tissues. Additionally, certain *CiTCP* genes displayed a high level of expression in particular tissues (FPKM > 20). For instance, *CiTCP15* and *CiTCP17* displayed high expression level in buds than other tissues, *CiTCP1* and *CiTCP8* presented high transcript abundance than other tissues, demonstrating that key roles of these genes in tissue development. It should be noted that some genes that were duplicated exhibited similar expression patterns. For example, *CiTCP3* and *CiTCP21* showed relatively high expression level in stem, while very low expression in other tissues. The expression levels of *CiTCP4* and *CiTCP17* were more elevated in bud, tongue flowers, and root compared to other tissues.

### Response of *CiTCP* genes to different abiotic treatments using RNA-Seq data

Cadmium (Cd^2+^), cold, hot and UV-B are common stresses that affect growth and reduce the production of *C. indicum.* To understand the potential functions of the *CiTCPs* in response to these different stresses, we extracted RNA-Seq data of the four stresses (cold, 4℃, 6 h, hot, 42℃, 6 h, UV-B, 24 h, Cd^2+^, 200 μmol, 24 h, listed in Additional file 7). According to the results, the *CTTCP* genes responded to cold and UV-B stress to a greater extent than to hot and Cd^2+^ treatments (Additional file 7 and Fig. 9). Among them, *CiTCP4, CiTCP15* and *CiTCP25* significantly induced against the cold treatment, while *CiTCP1* and *CiTCP26* were down-regulated. Under UV-B stress, most genes were significantly down-regulated, such as *CiTCP15, CiTCP24, CiTCP3, CiTCP8* and so on, *CiTCP4, CiTCP11, CiTCP17, CiTCP19* and *CiTCP25* were up-regulated. In response to hot stress, eight genes showed an increased expression patterns and 14 genes were more or less reduced, the remaining members could not be detected in hot response. We deducted these genes as pseudogenes or may be expressed only at specific developmental stages or under special conditions. For the Cd^2+^ treatment, *CiTCP4, CiTCP7, CiTCP17, CiTCP26* were significantly down-regulated, *CiTCP1, CiTCP5, CiTCP8, CiTCP12, CiTCP20* were up-regulated. Most members of the *CiTCP* family showed diverse expression patterns under different abiotic stresses. Interestingly, we found that *CiTCP25* was up-regulated under all treatments, suggesting that it might be a candidate gene for mitigating abiotic stresses.

### Validation of the expression profile of selecting *CiTCP* genes by qRT-PCR under abiotic treatments

**Fig. 10:**
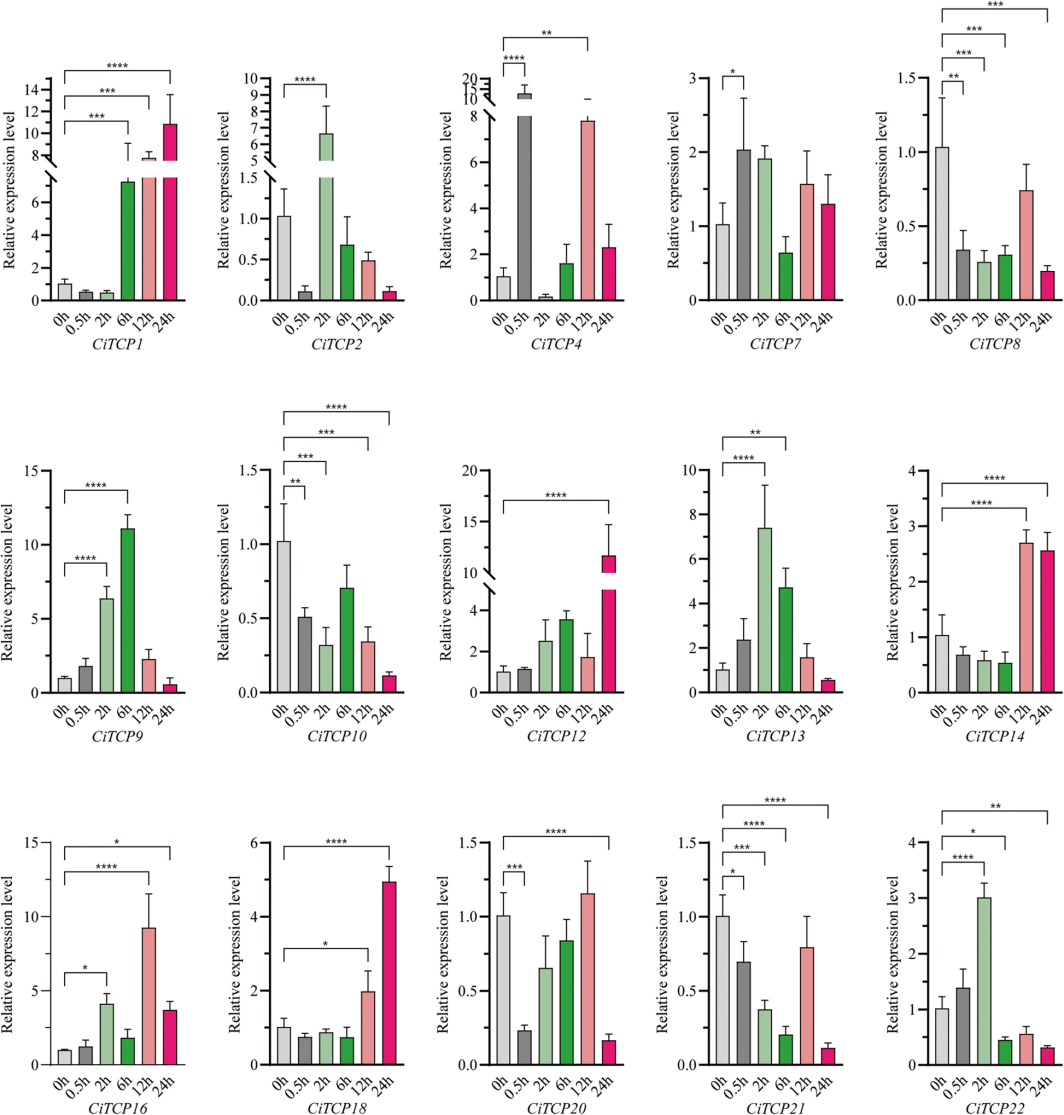
Expression pattern of *C. indicum TCPs* in response to cold stress determined by qRT-PCR. The Y-axis indicates the relative expression level and the X-axis represents different time points after stress treatment taken for expression analysis. The data presented are the average of three biological replicates, the bar represents the standard deviation

To delve deeper into the potential impact of different stress treatments on the expression of these *CiTCP* genes, a subset of 15 members from various subgroups were subjected to qRT-PCR (Fig. 10). The qRT-PCR results showed that most of the tested *CiTCP* genes were induced potentially under different stresses and the expression levels of these genes were correlated with the RNA-Seq data. Under cold stress, the tested *TCP* genes showed different expression patterns. For example, *CiTCP4* and *CiTCP13* were significantly up-regulated with 12-fold and twofold increase, while *CiTCP1*, *CiTCP2*, *CiTCP8*, *CiTCP10* and *CiTCP20* were down-regulated in response to low temperature treatment at 0.5 h, indicating these genes were regulated for a rapid response to cold stress. Compared with the untreated leaves, *CiTCP2/9/12/13/16/22* were dramatically up-regulated and *CiTCP1/4/8/10/14/21* were down-regulated after 2 h cold treatment, suggesting these genes may be cold acclimation genes. After 6 h treatment, which generated 4 genes (*CiTCP1/9/12/13*) were significantly up-regulated and 4 genes (*CiTCP8/14/21/22*) were down-regulated, suggesting that these genes may interact with other proteins to initiate a cascade of downstream signal pathway. *CiTCP1/4/9/14/16* were up-regulated and *CiTCP2/10* were down-regulated after 12 h cold treatment. *CiTCP1/4/12/14/16/18* were up-regulated and *CiTCP2/8/21/22* were down-regulated after 24 h treatment, indicating that these genes could be crucial for long-term cold acclimation.

**Fig. 11:**
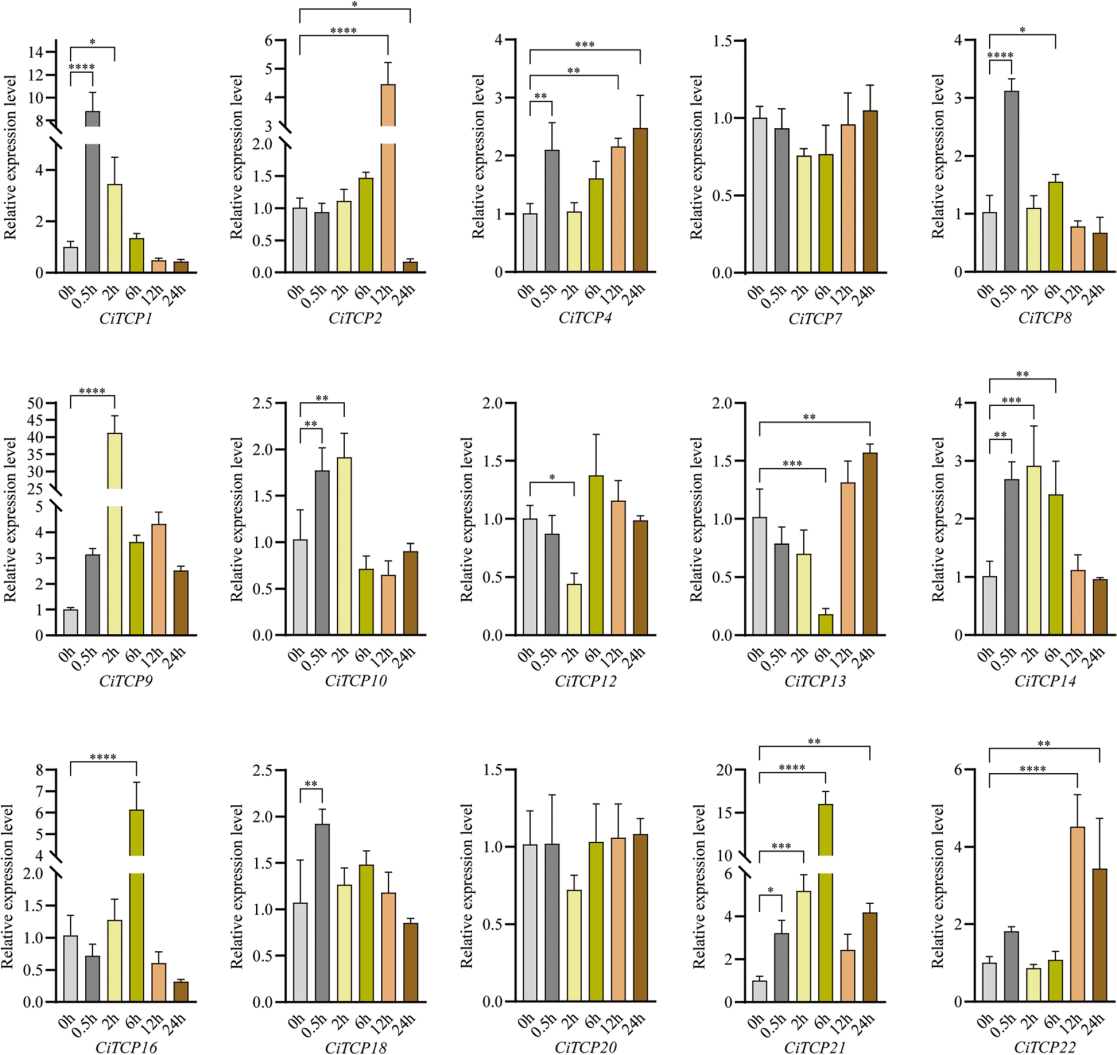
Expression pattern of *C. indicum TCPs* in response to drought stress determined by qRT-PCR. The Y-axis indicates the relative expression level and the X-axis represents different time points after stress treatment taken for expression analysis. The data presented are the average of three biological replicates, the bar represents the standard deviation

Under drought conditions (Fig. 11), nine members (*CiTCP1/2/10/21/8/9/14/16/18*) showed similar expression patterns, they each had a sharp increase and reached their peak value at early point, then had a down trend. For example, the expression of *CiTCP1* and *CiTCP8* increased at 0.5 h but as the treatment time increased, the expression level subsequently decreased. The expression of *CiTCP16* and *CiTCP21* reached the highest at 6 h and then decreased gradually. *CiTCP2* and *CiTCP22* reached the highest at 12 h. However, the expressions of *CiTCP12* and *CiTCP13* were significantly down-regulated at 2 h and 6 h, and then increased gradually. However, the expression level of *CiTCP7* and *CiTCP20* were not changed significantly.

**Fig. 12:**
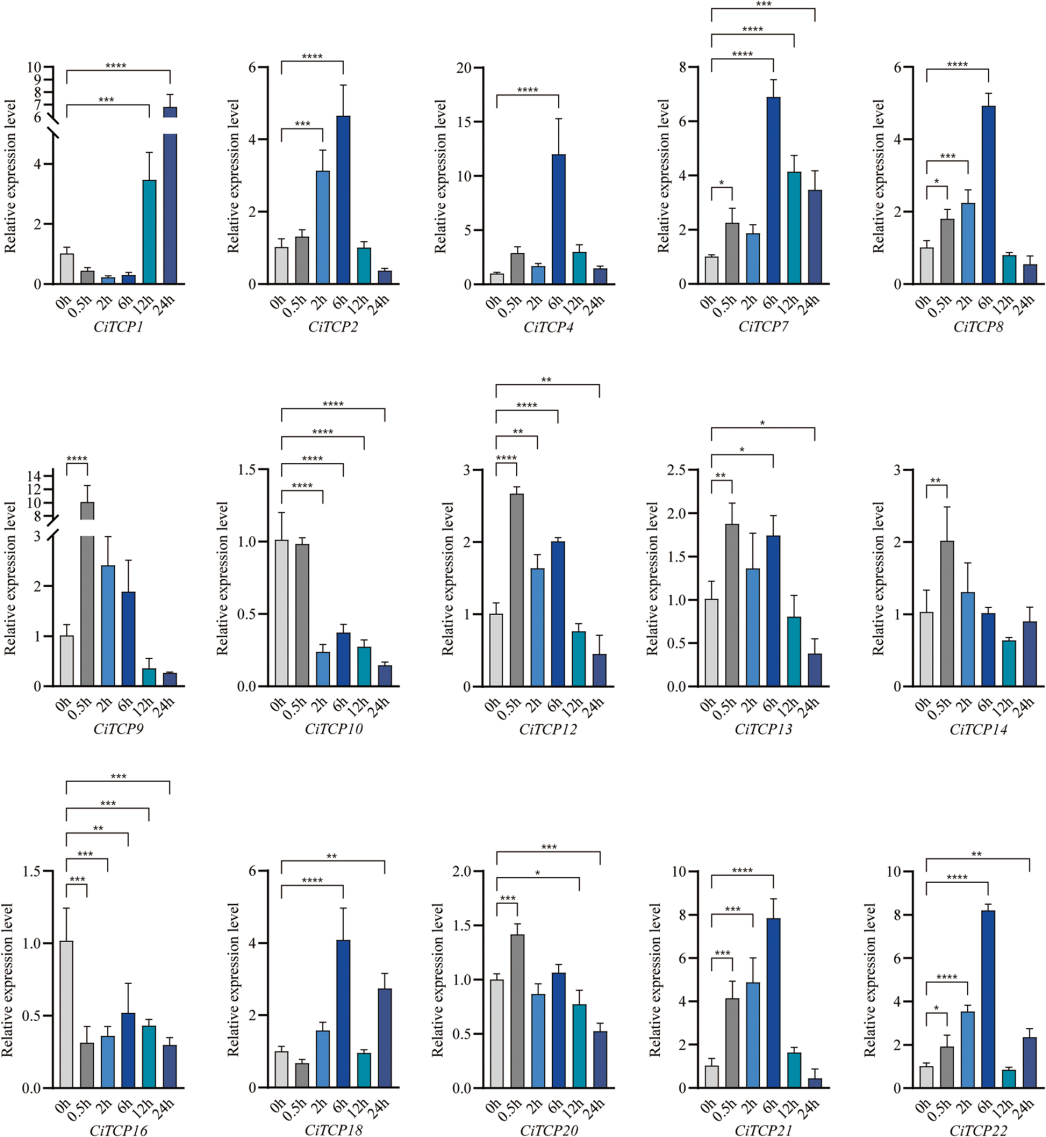
Expression pattern of *C. indicum TCPs* in response to salt stress determined by qRT-PCR. The Y-axis indicates the relative expression level and the X-axis represents different time points after stress treatment taken for expression analysis. The data presented are the average of three biological replicates, the bar represents the standard deviation

For the salt stress (Fig. 12), *CiTCP1* was down-regulated at early points, then increased and peaked at 24 h. *CiTCP16* and *CiTCP10* were down-regulated at all time periods. *CiTCP2/4/21/7/8/18/22* were all up-regulated and peaked at 6 h, with *CiTCP4* peaking 12-fold up-regulated of salt stress. *CiTCP12/13/9/14* were all up-regulated and peaked at 0.5 h, with tenfold up-regulated. *CiTCP1* which was down-regulated and then up-regulated. *CiTCP22* showed an up-regulated, down-regulated and then up-regulated expression pattern. NaCl had no significant effect on the expression levels of *CiTCP20*.

Collectively, the results confirm that these *CiTCP* genes might be involved in response to stress, which is consistent with the result of *cis*-acting element analysis in the promoters.

## Discussion

*C. indicum* is an important resource plant with high medicinal and ornamental value. *TCP* genes play important roles in plant developmental and physiological process, as well as various plant biotic and abiotic stress responses. In this study, we performed a comprehensive identification and analysis of *TCP* genes in *C. indicum*, with an aim of exploring the potential roles of this gene family in regulation of development and stress resistance in *C. indicum*.

Totally, we have reported a total number of 26 *CiTCPs* by complete genome screening of *C. indicum* using two search methods. There is a difference in the number of *TCP* gene family members between different species. A total of 26 *TCP* members were identified in the *C. indicum* genome, the size of gene number is closely related to *Arabidopsis* (24), *tomato* (30), *potato* (31), *O. sativa* (28), *P. trichocarpa* (36), but significantly differ with *strawberry* (19), *P. edulis* (16), *T. aestivum* (66), *P. virgatum* (42) [14, 19, 21, 23–29, 37, 38]. In general, there are two possible causes for the difference. Firstly, the size of the plant genome may affect the scale of the *TCP* gene family. As an illustration, *C. indicum* has a genome size of more than 3 Gb, and *T. aestivum* has a genome size of over 15 Gb. Despite having a genome that is over 1 Gb smaller than *C. indicum*, *P. virgatum* has a higher *TCP* gene count than *C. indicum*. The *TCP* family members in *Arabidopsis* and *O. sativa* are similar to those in *C. indicum*, despite having genomes of 135 Mb and 1.15 Gb. The duplication event is another factor contributing to the difference, and it could be the main reason for the expansion of the *TCP* gene family. *C. indicum* has encountered at most two WGD events. Likewise, *Arabidopsis* and *P. trichocarpa* have also encountered two and one WGD events, respectively [30, 39, 40].

Phylogenetic analysis (Fig. 1) and protein sequence alignment (Fig. 2) demonstrated that the 26 *CiTCP* members could be classified into two main classes (Class I and Class II) and three subgroups (PCF/CIN/CYC/TB1), which is similar with the classification of *TCP* genes in other species. We found that the homologous genes of 26 *CiTCP* genes were almost from either of *Arabidopsis* and *P. trichocarpa* (Fig. 1), indicating that *CiTCP* gene family has the evolutional conservation and closer homology relationship with closely related species. Furthermore, the distribution of *TCP* genes within each subgroup assumed that dicotyledonous plants occupy a larger proportion, suggesting that *TCP* genes underwent expansion from a common ancestor before angiosperm evolution and speciation. The *TCP* gene family featured by the conserved TCP domain, which forms a bHLH structure [7]. Be similar to the mechanism of the amphipathic helix (K region) in the MADS-BOX proteins, the bHLH domain may regulate protein–protein interaction in *TCP* genes. Also, most members in the CYC/TB1 clade possessed the R domain with unknown function might facilitate protein–protein interaction [6]. According to the phylogenetic analysis, *CiTCPs* shared similar motif compositions and gene structures were clustered together, further supporting the close evolutionary relationship among *CiTCP* genes.

Tandem, segmental, and whole-genome duplication are important sources of the functional diversity and evolution of gene families [37]. For detection of the expansion mechanism in *CiTCP* gene family, we first identified the tandem duplication and segmental duplication event. As shown in Fig. 3, most *CiTCP* genes distributed on chromosomes but clustered, there is no tandem duplication event was identified in these clusters with BLASTP method. In contrast, five segmental duplication events involving 26.9% (7/26) *CiTCP* genes were identified (Fig. 4 and Table 2). Also, all the *CiTCP* genes were segmentally duplicated within one subgroup to expand their members. Our results are largely similar with that described in orchard grass, also in *A. thaliana* and *O. sativa*, indicating that *TCP* duplication in plant genomes possibly has a common mechanism [26]. In *P. trichocarpa TCP* gene family, 13 pairs of duplicated genes were identified, accounting for about 72% of the *P. trichocarpa TCP* family, and they result from segment duplications rather than tandem duplications [24]. For the *switchgrass*, no tandem repeats occurred in the evolutionary process in *switchgrass TCP* genes, the large enrichment of *switchgrass TCP* genes was presumably due to the allotetraploid event [28]. The duplication mechanisms vary among different species, which suggests that different duplication events played a different role in the history of evolution. Comparative mapping (Fig. 5) established the orthologous and paralogous relationship among dicotyledonous and monocotyledonous species. Interestingly, the presence of 39, 24, 7 and 7 collinear pairs between *C. indicum* with *G. max*, *V. vinifera*, *A. thaliana* and *P. trichocarpa*, respectively, but only one pair in *O. sativa*, may suggest that these orthologous pairs may be involved in divergence of dicot and monocot plants. Different species have different collinear pairs with *C. indicum*, which suggests that these orthologous pairs were created during the divergence between dicot and monocot plants. The high conservation of TCP proteins across different species suggests that *C. indicum* shares significant functional similarity with other plants. This suggested that *TCP* genes could be closely related to different species, along with similar biological functions. In terms of every subgroup, the PCF subgroup contained the most segmental duplication events with three pairs, but the expansion of gene number in this subgroup was small, almost comparable to the other species in the phylogenetic tree (Fig. 1 and Table 2). In contrast, the CYC\TB1 subgroup only possessed one pair duplication event, but the *CiTCP* genes in this subgroup were larger than other species. In the remaining subgroup, the number of *TCP* members in *C. indicum* was less than or equal to that in *Arabidopsis*, *O. sativa* and *P. trichocarpa*. Indeed, the Ka/Ks ratio results showed that the duplicated *CiTCP* genes were under strong purifying selection.

Expression pattern analysis could provide more insights into the potential roles of the *TCP* gene family. According to the qRT-PCR results (Fig. 10), the 15 selected genes from different groups showed different expression patterns under different stress treatments. For example, the expression of *CiTCP10* was enhanced by drought but was declined under salt and cold stress. Also, the expression of *CiTCP8* was down-regulated under cold stress, while it was up-regulated by salt and drought. These results suggest that *CiTCP10* and *CiTCP8* might have different mechanisms to maintain protection against various abiotic signals. We also found that some *CiTCP* genes were responsive to specific stresses. For instance, *CiTCP13* was significantly induced by drought and cold, but not by salt. *CiTCP18* was significantly induced by salt and cold, but not in drought. Interestingly, most members in the same subgroup showed different expression patterns. For instance, *CiTCP10/14/16*, belong to the CIN subgroup, *CiTCP10* and *CiTCP16* were significantly down-regulated by salt stress, while *CiTCP14* were strongly induced. *CiTCP2/4/8/9*, belong to the PCF subgroup, *CiTCP2*, *CiTCP4*, and *CiTCP9* were up-regulated by cold stress, and in contrast, *CiTCP8* were down-regulated. Moreover, we examined the expression patterns of duplicated genes in different tissues. The results indicate that TCP genes that are clustered in segmental duplication pairs generally have similar expression patterns. For example, the segmental duplication pairs, *CiTCP3* and *CiTCP21* were abundantly expressed in stem, *CiTCP15* and *CiTCP17* were characterized by high expression in leaf and tongue flowers. The analysis of functional genes in *C. indicum* revealed that these genes play various roles in different stress conditions.

TCP proteins orchestrate a lot of plant developmental processes and respond to environmental stimuli. According to the prediction results of *cis*-regulatory elements in *CiTCPs* promoter, we found that *TCPs* contained many elements involving in abiotic stress response, such as, *CiTCP5*, *CiTCP14* and *CiTCP24* possessed DRE element involved in dehydration, low-temperature, salt stresses, more than half of *CiTCP* genes contained LTR element, suggesting that *TCP* genes play a significant role in stress response. For example, *CiTCP25* contained LTR, MBS and TC-rich repeats elements in promoter, the expression of *CiTCP25* were induced more than threefold after cold treatment. Also seen in *CiTCP9*, one TC-rich repeats element exhibited in promoter region, the expression changed more than 3 times compared to the 0 h. In *G. max*, most *GmTCPs* appeared to respond to heat, cold, and defense stress, which was similar with the results in *C. indicum* [38]. Stress-responsive *cis*-acting elements were present in all 26 CiTCP genes, indicating their potential roles in response to abiotic stresses (Additional file 5 and Fig. 7). It has been proven by many studies that TCP genes are involved in responding to various plant abiotic stresses. For instance, overexpression of *ZmTCP42* in *Arabidopsis* led to a hypersensitivity to ABA in seed germination and enhanced drought tolerance. Here, qRT-PCR was used to analyze the expression of *CiTCPs* under multiple stress treatment (Fig. 10). The results showed that *CiTCP2/9/14/16/21/22* were significantly induced against salt, cold and drought conditions, and all of them contained DRE, LTR, MBS and TC-rich repeats elements in their promoters, indicating that these two genes may integrate different stress signals. The functional information of *CiTCPs* can be predicted according to their identified orthologous in *Arabidopsis* and *Rice*. According to the PPI prediction (Fig. 8), CiTCP2/10/14/16/22 may interact with SAP11, and participatein many abiotic stress responses. Also, CiTCP10/14/16 belong to the CIN subgroup, CiTCP10 shared homology with AtTCP4. Previous studies showed that *TCP4* may play crucial roles in plant resistance to abiotic. In addition, *TCP4* targeted by miR319 s is involved in abiotic stress response such as high salt and drought have been demonstrated [39, 40]. This suggests the potential involvement of *CiTCP10* in multiple abiotic stress responses and warrants further exploration of its specific functions in these conditions. *CiTCP8*, had homology with *OsTCP19*, belonged to the PCF subgroup, and was highly induced under salt and drought stress. Overexpression of *OsTCP19* in *Arabidopsis* could improve the drought tolerance and the overexpression of *GmTCP4* can increased the drought tolerance in *soybean* [18]. So, we suspect that *CiTCP8* may also take part in the drought response in *C. indicum*. Verification of the initial findings requires further study of the results. Analyzing the biological functions of these genes will provide a significant theoretical basis for improving *C. indicum* quality.

## Conclusions

In this study, 26 *TCP* genes were identified through comparative genomics analyses of sugarcane. Systematic informatics analyses were performed, including phylogenetic, conserved motif and intron/exon analyses. Duplicated analysis revealed that segmental supplication event contributed to the expansion of the gene family, and purifying selection was the main force driving evolution. Moreover, the expression levels of *CiTCPs* were analyzed in response to various treatments (UVB, Cd^2+^, hot, cold, drought, NaCl) based on available RNA-seq data and qPCR validation, indicating that *CiTCPs* played important roles in *C. indicum* stress tolerance. Furthermore, our findings provide the foundation for further functional characterization and identification of the regulatory mechanism of *TCP* genes in plant stress responses. Additionally, candidate *CiTCPs* can be employed for germplasm improvement using molecular breeding techniques and genome editing tools to enhance production.

## Supplementary Information

Additional file 1. Phylogenetic tree used NJ method representing relationships among TCP gene family of C. indicum. The different colored areas indicate different subgroups respectively.

Additional file 2. Multiple alignments of R domain in CiTCP proteins.

Additional file 3. Synteny blocks of TCP genes between C. indicum and Arabidopsis, rice, soybean, populus and grape genome.

Additional file 4. Ten predicted conserved motifs of the 26 CiTCP proteins.

Additional file 5. Details of the cis-acting elements identified in this study.

Additional file 6. The GO enrichment analysis of TCP genes in C. indicum.

Additional file 7. RNA-seq data of the CiTCP genes in different tissues and treatments.

Additional file 8. Primers used in quantitative RT-PCR of CiTCP genes.

## Data Availability

All relevant data are within the manuscript and its Additional files.

## Abbreviations

TFs: Transcription factors
qRT-PCR: Quantitative RT-PCR
TCPs: The Teosinte Branched1/ Cycloidea/ Proliferating Cell Factors
bHLH: Basic helix-loop-helix
BR: Brassinosteroid
CAT: Catalase
H_2_O_2_: Hyderogen peroxide
ROS: Reactive oxygenspecies
ABA: Absdsicacid
HMM: Hidden Markov Model
ML: Maximum likelihood
kb: Kilobyte
MCScanX: Multiple Collinearity Scan toolkit
Ka: Non-synonymous rate
Ks: Synonymous substitution rate
GO: Gene Ontology
PPI: Protein-Protein Interaction
RNA-Seq: RNA-sequeencing
FPKM: Fragments-per-kilobase-per-million
UV-B: Ultraviolet radiation b
PEG: Polyethylene glycol
M: Mol/L
RNA: Ribonucleic Acid
PI: Theoretical isoelectric point
kDa: K Dalton
N-J: Neighbor-joining
ML: Maximum likelihood
CDS: Coding sequence
UTR: Untranslated region
Cd: Cadmium
Gb: Gigabyte
WGD: Whole Genome Duplication
