# Supplementary figures and images for: Genome-wide identification and expression analysis of TCP transcription factors in Chrysanthemum indicum reveals their critical role in the response to various abiotic stresses
Source: BMC Plant Biol. 2025 May 13;25:631. doi: 10.1186/s12870-025-06521-x (PMC12070562; doi:10.1186/s12870-025-06521-x)

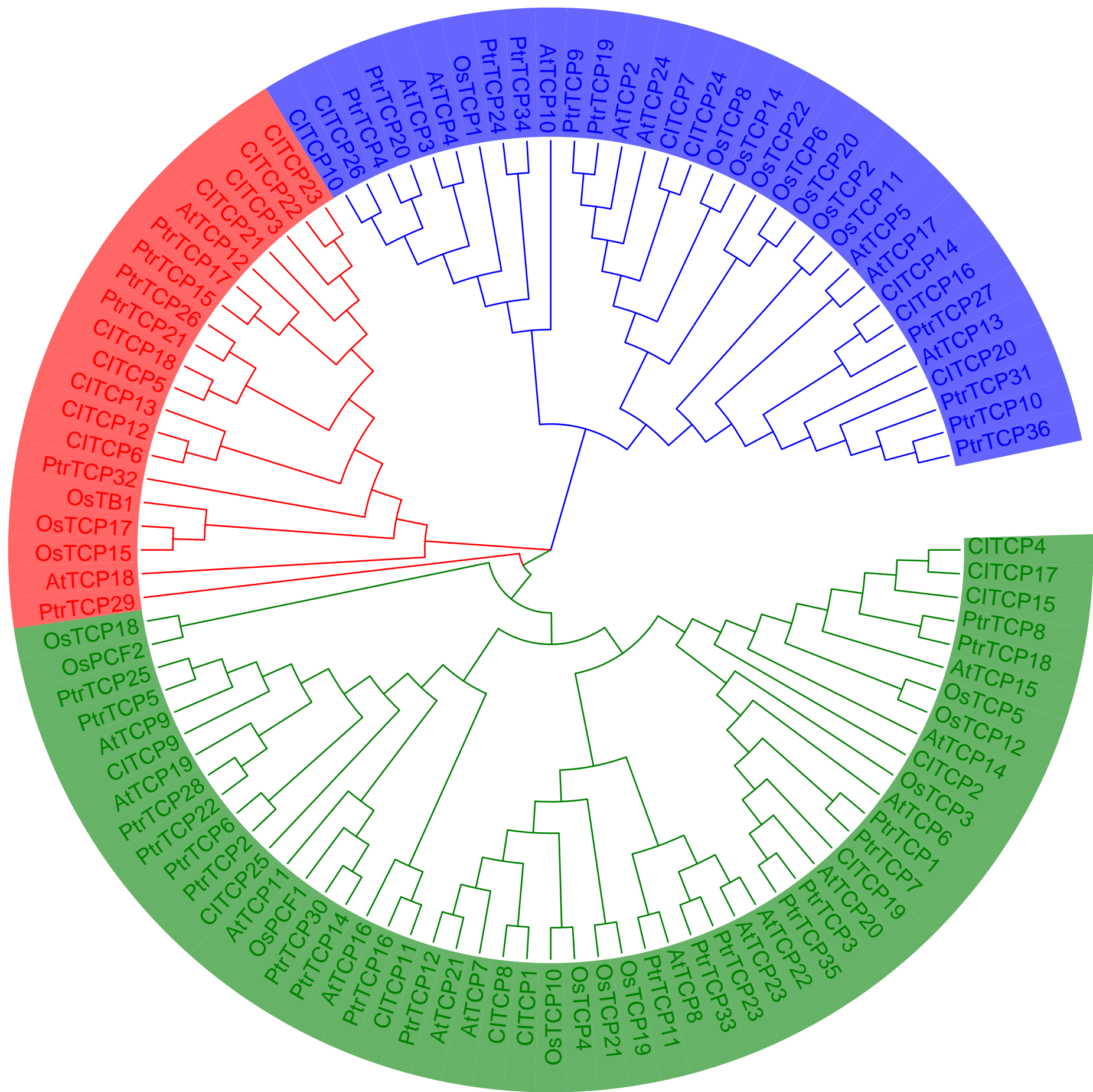

Supplement: Supplementary file 1 — Additional file 1. Phylogenetic tree used NJ method representing relationships among TCP gene family of C. indicum. The different colored areas indicate different subgroups respectively. [file 12870_2025_6521_MOESM1_ESM.pdf]
