# Supplementary material for: Genome-wide identification and expression analysis of TCP transcription factors in Chrysanthemum indicum reveals their critical role in the response to various abiotic stresses
Source: BMC Plant Biol. 2025 May 13;25:631. doi: 10.1186/s12870-025-06521-x (PMC12070562; doi:10.1186/s12870-025-06521-x)
Supplement: Supplementary file 2 — Additional file 2. Multiple alignments of R domain in CiTCP proteins. [file 12870_2025_6521_MOESM2_ESM.pdf]

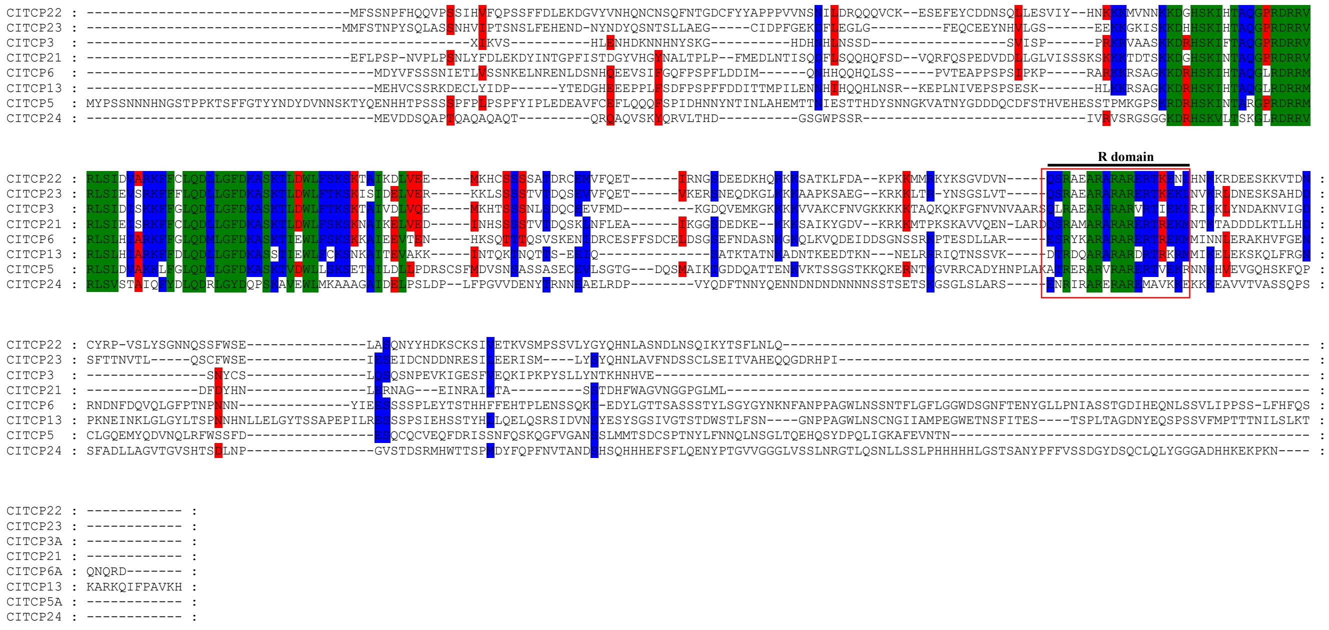
R domain
CITCP22
CITCP23
CITCP3
CITCP21
CITCP6
CITCP13
CITCP5
CITCP24
CITCP3A
CITCP6A
CITCP5A
